# Physiological Incompatibility of the Respective Sexes of Our Species*I do not mean by species the whole race, but a portion of it only.

**Published:** 1863

**Authors:** W. Byrd Powell

**Affiliations:** Covington, Ky.


### CHICAGO MEDICAL EXAMINER.

N. S. DAVIS, M.D., Editor.

VOL. IV. MAY AND JUNE, 1863. Nos. 5 & 6.

### Original Contributions.

ARTICLE XIII.

### PHYSIOLOGICAL INCOMPATIBILITY OF THE RESPECTIVE SEXES OF OUR SPECIES.*

By W. BYRD POWELL, M.D., Covington, Ky.

Messrs Editors:—Having made, as I believe, and as the editor of the *Scalpel* for January, '59, said, "the most important discovery ever made in physiological science;" and having reduced it to a highly practical simplicity, and being anxious that the sons and daughters of my country shall have the benefit of it as soon as may be practicable, I send for your *Journal* a brief exposition of it. As the history of a discovery, generally, furnishes the evidence, at least, of its probability, I will, therefore, make the history of this discovery my introduction.

In October, 1844, I was travelling in the State of Mississippi, and was taken sick on the road, so sick as to deem it prudent that I should seek some halting-place where I could be properly cared for, and, presently, I arrived at a small village, and drove to the door of a dry goods store and called for the proprietor. He appeared at the door, I informed him of my condition and desire to find a place where I could be properly cared for. He

* I do not mean by species the whole race, but a portion of it only.

responded, we have in this little community one of the best families known to this or, probably, any other country, and if they will receive you, and I think they will, you will not want for anything that an enlightened goodness can command. He indicated the house, I drove to it, and, luckily, I was received, for at a glance I perceived that the representation of the merchant was correct,—for the entire premises indicated the abode of plenty, comfort, and happiness. The host and hostess were in harmony with the surroundings; in personal health, development, and social kindness they were model representations of humanity. With all this, I did not feel entirely at ease. I saw no children, for I imagined their children, if any, to be miniature models of humanity. At length I ventured to ask the lady, if she had any children? She responded affirmatively, and added, "they are at school." In the evening, three little boys came in; and never have I been so much disappointed as I was at the sight of these children.

They were, respectively, afflicted with tabes mesenterica, in an advanced stage, and hence they were disgusting objects. The mother, I think, must have perceived my disappointment, and hence remarked: "my first three children are dead." Of what disease did they die? I inquired. She answered: "the first died of consumption of the lungs, and the other two of the bowels." As the parents had every indication of having a sound constitution and excellent health, and from the excellence of their residence and plentifulness on their table, I felt fully assured that their children had not been stinted in food, or exposed habitually, at any time, to a cold and humid atmosphere; and further, the country was exceedingly healthy, and believing at that time, as every body else did, that the consanguine relation of progenitors is mischievous to progeny, I asked the lady if she and her husband were cousins? She answered, "no, sir; there is no blood relation between us, and further, consumption was never known to have obtained in either his family or mine, and hence we are greatly surprised that our children should die of it." This case greatly puzzled me, and hence, during my sojourn with this family, I investigated every thing and circumstance that could apparently hold any relation to them, but finding no apparently possible cause for the scrofula of their children, I was forced to the suspicion that it resulted from some physiological condition in the progenitors which was yet to be discovered, and I believed that it could be discovered by a sufficiently extensive observation of parents and children, and I resolved to make an effort to discover it; consequently I made a memorandum of all the obvious facts which this case furnished, including the temperaments of the parents, respectively. But I did not suspect that they had anything to do with it, because I regarded them as normal conditions, but I noted them simply as a circumstance, and because to do so would help my memory, for at this time, and for the eighteen previous years, the temperaments had, with me, in a great measure, been a specialty, and probably I was more practically familiar with them than any other individual ever was.

The parties were, respectively, of the *sanguine*, *bilious*, *lymphatic* temperament; and, to the extent of my ability to judge, I never saw a more physiological couple.

The weather being fine and my health restored, I started on my journey. About one o'clock I halted at an inn on the road side, for dinner. The host was a fine and soundly looking representative of the bilious encephalic temperament, and the hostess was the same,—a superior woman. I asked the host, if he had children? He answered: "I have." I informed him that I was making observations on parents and children, and would therefore be pleased to see his. He responded: "certainly, sir," and led the way to the nursery. Upon entering it, I saw two children; one was rachitic and the other imbecile. On leaving the nursery, he remarked: "our first child died of brain fever." From the condition of the two I saw, I suspected that the brain fever of the first was of the scrofulous variety. Upon returning to the parlor, I found the mother weeping over her maternal disappointments, and I sympathized deeply with her, because she was sufficiently endowed to have been the mother of statesmen. These parties assured me that there was no consanguinity between them, and that scrofula, in no form, had ever, as they thought, been in the ancestry of either of them. Their circumstances indicated a comfortable independence, and, like the preceding family, they were sound, healthy, and lived in a healthy country.

Having dispatched a good dinner, I continued my journey, with my mind thoroughly perplexed. I meditated myself into fever and cephalagia, and hence, at an earlier hour than usual, arriving at an inn, I halted for the night. At my arrival the host appeared. His temperament was sanguine, bilious, which I regarded as being the most dense and enduring known to the species. The evening being pleasant, I seated myself on the piazza, and presently the hostess and seven children appeared, for my entertainment, possibly. Her constitution was bilious lymphatic; all the children had a sound, viable appearance, and the mother informed me that she had lost none. I found nothing here that appeared likely to aid in relieving my perplexity. My observation here, however, was not unprofitable. For it showed that for the production of a sound and viable progeny, that both progenitors need not have, in the abstract, the best constitution, for I did not think very favorably of her constitution, it was too watery. I now inquired of the hostess if she knew of any family who had been very unfortunate with their children. She answered: "I do. There lives a family ten miles distant from here, on the road you are traveling. They keep private entertainment, and you can easily reach there for breakfast, and you will get a good one." Although I obtained nothing in this family that promised to be of any avail, nevertheless, I made a note of it, as I intended to do of every family I saw, for otherwise I could not arrive at a profitable generalization.

Next morning I drove ten miles for breakfast. The host and hostess were, respectively, sanguine encephalic, had had seven children, and they, respectively, died at about the age of two years, some of them with hydrocephalous and the others of brain fever,—tuburcular meningitis, I suppose, because I now know that the children of such parents very generally die with the two forms of disease above-named. These parties were not consanguine, nor had they any suspicion of the cause of their misfortune. The subject now began to appear to me more inscrutable, and my suspicion of the cause being physiological was increasing, which made me more resolved to pursue the inquiry. These were interesting people, and hence, I more than usually enjoyed my breakfast with them. I was soon on the road again. I continued my drive till one o'clock, when I halted for dinner. The host was a strongly marked representative of the *bilious* temperament, and his wife was an equally well-marked representative of the *sanguine*, *bilious*, *lymphatic* temperament. They had three sound, healthy, and promising boys, had lost no children. Dinner being dispatched, I was again on the road and drove till dark; but, finding no hotel, I claimed the hospitality of a planter, and it was cheerfully granted. He was a splendid representation of the *sanguine*, *bilious*, *lymphatic* temperament, and his wife was an equally fine representation of the *sanguine*, *bilious*, *encephalic* temperament, and a lady. I had a very pleasant evening with them. They had had six children, but all were dead; some died of tabes mesenterica, one of phthisis, some of brain fever, and the youngest, an infant, of croup. Between these parties there was no consanguinity, both claimed to be sound and healthy, and so they appeared to be. They seemed confident that no scrofulous form of disease had been in their respective ancestors. They had no suspicion of the cause of their loss.

On the next morning I made a tolerably early start, and drove ten miles for breakfast; and found the host and hostess to be more than usually intellectual and interesting, with, apparently, sound constitutions and good health. They were, respectively, fine illustrations of the *bilious encephalic* temperament, had been married a little more than twenty years, but were still childless; and between them there was no consanguinity. Every thing about this house conspired to fit me for the road,—the day was pleasant and beautiful and the road good; and, hence, almost as soon as I was again on the road I, spontaneously, began to meditate on the observations I had made, and noted the number, and found it to be seven. Indeed, in memory, I re-travelled the road and visited each family; and, by generalizing them, I saw that the seven families, that is, the parties to them, possessed similarly good constitutions and health, that they lived similarly well, and in a similarly healthy country, and, further, there was no consanguinity between the parties, respectively.

This review shed no light on my difficulty. I now reviewed and generalized their temperaments,—a matter to which, at first, I attached but little importance. I found between the third and fifth parties, respectively, a very considerable dissimilitude of constitution, and that their children, respectively, were healthy, and of viable promise. But between the first, second, fourth, sixth, and seventh parties, respectively, there was a very close similitude of constitution; and, with reference to progeny, they had all been unfortunate.

Indeed, the first, second, fourth, and seventh parties respectively were the same. The parties to the sixth differed nominally a little, but reproductively or vitally they were the same, for, several years before this event, I had concluded that the lymphatic and encephalic temperaments were equivalent in relation to the vital functions. Hence, so far as the generalization of seven cases could warrant an inference, it must be that physiological similitude between the respective sexes of our species renders them incompatible in relation to the procreative functions; but for several years I did not allow this inference to have any other effect on me than that of a suggestion, but more than eighteen years of subsequent observation has confirmed that inference as being the most important truth ever announced in physiological science. This discovery has revealed to me the remote cause of the scrofulous diathesis of the long charged *opprobrium medicorum.* Thus, in two days and a fraction after resolving to make an important discovery in the most occult department of anthropology, I succeeded.

The human temperaments were introduced to the attention of the medical profession more than two thousand years ago, and how it has happened that the fact of the very frequent existence of a physiological incompatibility between the most physiologically sound and healthy parties of the respective sexes, was not discovered many centuries since, is a question which I cannot solve, without assuming that the profession has not given them that attention they merit, and this I believe to be the fact. For who is, or has been, the physiologist who could authenticate even the simple temperaments, and much less their compounds, by an inspection of denuded crania? If such an one ever lived, I have not become informed of it, and yet this is so possible that every physiological professor should be capable of it.

Since making this discovery, I have consulted every authority I have been able to command, to ascertain whether I had been anticipated; but have not found the most remote intimation that a physiological incompatibility ever obtains between the sexes of any zoological species, with the exception of one recent writer, Dr. T. L. NICHOLS, who, in a small volume entitled *Eroteric Anthropology*, published in New York in '53, between eight and nine years subsequently to the date of my discovery. He announces it to be a fact, that a physiological incompatibility does obtain, occasionally, between the respective sexes of our species, and denominates it "physiological incest." He thus very clearly indicates the character of this incompatibility. He does not, however, indicate the conditions that conspire to produce it, nor the indices of the fact when it does obtain. His statement may therefore be nothing more than the expression of an hypothesis, which appears to be instinctively founded in the popular mind.

But however this may be, before the date of his publication by several years, I had not only discovered the fact, but also the conditions that produce it, and also its indices; and consequently, at the sight of incompatible parties of the respective sexes, whether married or single, I was able to announce the fact, and, in many cases, the consequences, with a scientific certainty.

The existence of a physiological incompatibility between the respective sexes of our species, has, virtually, been conceded to obtain, possibly for centuries, between consanguine parties; but this, I am confident, is either an error or a prejudice. I admit, however, that consanguine parties are occasionally unfortunate as progenitors, but the cause is not consanguinity, but that which frequently obtains with other parties. Of the seven marriages I observed in making this discovery, two of them only, as I now know, were physiological; and I think it highly probable that not more than two-sevenths of our marriages, generally, are compatible or physiological; and as the consequences of incompatible marriages are sterility, scrofulously constituted children, imbecile, blind, and deaf children; we have plainly the source of our many sterile marriages, scrofulous forms of disease, asylums for imbecile, blind, and deaf children.

The fundamental fact of sexual incompatibility in our species is, physiological similitude, and it attains in the union by marriage of certain of those conditions which are denominated the human temperaments; consequently, for the purpose of being clearly understood, it becomes indispensable that I should treat, to some extent, of the temperaments.

By temperament, I mean a *sui-generis* mode of human life, compatible with health and longevity. With an essential modification, I adopt the Hippocratic system, which comprises four conditions, and which have been assumed to be elementary, viz.: *the sanguine, the bilious, the lymphatic, and the melancholic.* But having, in common with other physiologists, concluded that the last is a condition that has not and never had a physiological existence, I discarded it. I never doubted, however, that Hippocrates did observe a fourth condition; and observation has forced upon me the conviction, that humanity comprises four conditions; and this conclusion is rendered certain by the fact, that the other three do not constitute a system, and, therefore, do not furnish the elements of the compound conditions which numerously obtain in all communities. The truly fourth temperament I claim to have discovered in 1832; and this so thoroughly perfects the system, that neither myself nor pupils have any difficulty in distinguishing the combinations. I do it readily with denuded crania. I am now entirely confident that the ancients approximated the discovery of this temperament, that their melancholic was a compound of the bilious with the one I discovered, with, probably, a pathological condition of the portal system.

The one I discovered I denominated the Encephalic temperament; and as your readers have not, probably, seen the work entitled, "The Natural History of the Human Temperaments," by the writer, it is proper that I should indicate the principal indices of the encephalic temperament:—Like the lymphatic, physiologists to the contrary notwithstanding, the encephalic has no diagnostic or distinguishing complexion, it may be either fair or dark. In this temperament the cerebrum, in relation to the cerebellum, is large, and, of course, the cerebellum, in the same relation, is small, and, consequently, all the vital functions, except absorption, are feebly and tardily manifested; the thorax and abdomen are small and contracted; the muscles are slender, feeble, and flaccid; the locomotion is dragging; the neck is long and slender; the anterior lobes of the cerebrum are massive, prominent, and superiorly expanded. People of this class are capable of profound thought and motion, but not of powerful, and are greatly liable to monomania.

I think it probable that no amount of observation my readers can command would enable them to see a strongly defined representative of this class, especially, in our country; but they may observe it in combination with the other temperaments, in almost every alternate person they meet in our cities, particularly; and its presence is indicated by an expansion of the superior third of the forehead.

I have reduced this discovery to a highly practical science, which I denominate *the science of physiological marriage*, and it affords the only reliable guide to a physiological marriage, or one that will not be productive of evil consequences to progeny; and, fortunately, it is so simple that any clever Miss of ten summers can, by the aid of a capable instructor, become practical in the application of it in three or four weeks, and without such help, scarcely one per cent. of our most intelligent people could acquire the same ability in the half of a long life. In treating of this science, I have found it useful to divide the four elementary temperaments into two classes, the *vital* and the *non-vital;* the former comprises the sanguine and the bilious, and is so denominated because observation has forced on me the conviction, that without the agency of one or the other of them, there can be no transmission of life. The latter comprises the lymphatic and the encephalic, and is so denominated because as frequently as I have observed the respective parties to a marriage to be as much as two-thirds of these conditions, so frequently have I learned that three-fourths of their children were dead-born, and the other fourth did not, respectively, live one year.

The vital temperaments I regard as having been founded originally in the constitution of humanity. But the non-vital I hold to have resulted from influences incidental to civilization, thus: it is conceded that wealth resulted from civilization, and wealth induces ease, idleness, and many varieties of indulgence, and these induce debility and lymph in a vital constitution, but more particularly in a humid atmosphere. This condition is not found among savage people, nor among a frontier people; and further, in the preceding thirty-five years, I have observed many sanguine, bilious, and sanguine bilious people to become, in a few years, comparatively, considerably lymphatic, under the influence of ease, idleness, and their associated indulgences; and when this condition becomes induced, no matter to what extent, a lymphatic diathesis becomes entailed, and thus rapidly disseminated.

Mental activity, care, responsibility, and sedentary habits, are about as exclusively incidental to civilization as wealth is, and they, by developing the cerebrum to the neglect of the cerebellum, induce the encephalic condition. This temperament, like the preceding, is not to be found with primitive and frontier people, and when induced it is disseminated by entail. I have frequently observed this condition to be rapidly developed in young men holding responsible situations in banking and commercial houses. It must have been noticed that these non-vital conditions are not exclusively elementary because they are respectively founded on a vital or elementary condition. They are therefore merely adjunctive—induced modifications of vital or elementary conditions.

It must be understood that these adjunctive conditions do not acquire the consideration of temperaments, until they become so developed as to obliterate the indices of the fundamental condition, except the complexion. It must now be understood why it is that these conditions respectively have not a diagnostic complexion. For if founded on the sanguine condition, the complexion will be fair, but if on the bilious, dark, etc.

It is now seen that there are but two original or primary temperaments, if the preceding views be sound, and so I regard them. Nevertheless, as the non-vital conditions observe the laws of combination, that the others do as they, respectively, exert a specially dynamic influence over the constitution and the mental manifestations, I deem it best to regard and treat them as temperaments. These conditions are of great importance to civilized man,—they greatly increase the varieties of character and useful instrumentalities; but when they become, numerically, greater than the vital conditions, the tendency of the species is toward extinction; and the same is true when the vital conditions acquire a numerical ascendency. Hence, in the present constitution of the species, both classes are indispensable to its prosperity; that is, neither class would alone perpetuate the species.

It is a remakable fact, in the physiology of the pro-creative functions of humanity, that an extreme similitude, and an extreme dissimilitude of constitution between the respective sexes renders them equally incompatible. For both cause the parties to be either sterile or to entail on their progeny a scrofulous diathesis. As extreme dissimilitude cannot obtain between parties who are, respectively, of the same species, but when they are of remote species, as, when one party is of the white, and the other party is of the negro species of our genus, in such alliances, the progeny is, invariably, scrofulous, I believe. It is, therefore, only of the former branch of the fact of which I now propose to treat.

That a considerable dissimilitude of constitution between the respective parties to a marriage is essential to the production of a sound and viable progeny, is a fact that admits of no disputation; and, indeed, an impression of this truth is, instinctively, founded in the human mind, as is shown by the fact, that people contemplating marriage prefer a difference or contrast between themselves, respectively, and the party wanted. This instinct is right, but, before it can be trusted, it must be enlightened. In this relation, I present the following laws:—

FIRSTLY.—*When the respective parties to a marriage are so nearly similar that a qualified observer cannot detect any appreciable difference between them, sterility will be the result.* Illustration.—General Washington and his wife were, respectively, sanguine, and sterility is known to have been the result; and General Jackson and his wife were, respectively, bilious, and and sterility is known to have been the result. The first Napoleon and his first wife though, apparently and nominally, they were dissimilar, and yet, physiologically, they were very similar. His constitution was *sanguine*, *bilious*, *encephalo-lymphatic*, and she was *bilious-encephalic;* hence, they were, respectively, compounded of equal moieties of vital and non-vital conditions, and their marriage was sterile.

SECONDLY.—*When the constitutional difference between married parties is slight, but appreciable, progeny may result, but it will be either imbecile, scrofulous, deaf, blind, or unfortunate in some other respect.* The second wife of Napoleon was sanguine encephalo-bilious; hence, in having none of the lymphatic, it differed from his,—her constitution was one-third non-vital, and his was one-half; this difference brought them a son, but the difference was too small to save him from a scrofulous diathesis and a scrofulous death before adult age.

THIRDLY.—The marriage most favorable to a sound and viable progeny, is that in which the parties are, constitutionally, as dissimiliar as it is possible for them to be, within the limits of the species,—that is, one party is exclusively vital and the other is as non-vital as possible, as, when one party is sanguine and the other is lymphatic or encephalic. But as such marriages are greatly impractical in any country, and as a less degree of dissimilitude is productive of a physiological marriage, I found it to be essential that I should find the law that covered all degrees of physiological marriage, that is, all marriages compatible with a sound and viable progeny; and that law is

Fourthly.—*One of the parties must be vital, that is, sanguine, bilious, or sanguine-bilious,* (the last, being a compound of the two former, is also viable, and may be substituted for either of them,) *and the other party must be more or less non-vital, and more the better, and less than one-third I cannot advise.* Those who make a viable progeny a condition of marriage must conform to this law, and all infringements of it will be attended with evil consequences. It will now be seen that the marriages of Washington, Jackson, and Napoleon were, respectively, violations of the fourth law.

I will conclude by presenting a few cases of my practice in this relation, out of about three hundred of which I have memoranda, to illustrate the truth of the subject and its practical application.

In 1848, in Oxford, Miss., Mrs. ——— called on me and said: "Prof. Powell, by my attention to your lectures, my curiosity to have your opinion of my marriage has been excited. This, sir," handing me a little box, "is a daguerreotype of my husband." After directing my attention to it, I responded: I think your marriage has been unfortunate. She rejoined: "how unfortunate?" I answered, you have had no offspring, I think; but if — she interrupted by saying: "you have no need of any buts or ifs, for you have fully answered my question. I have been married more than eighteen years, but am still childless."

These parties were respectively *bilious encephalic.*

In 1849, at Raleigh, Tenn., Mr. ———, a planter, called on me and said: "Professor, I heard your lecture last night, and as regards children, I have been rather unfortunate. I have called with a portrait of my wife to obtain your opinion of my prospect of children by her." I responded: as both of you have a sound and healthy appearance, I think your prospect of children is very good, but they will not be such as either you or she will desire. He rejoined, "why not, sir?" I answered, because, they will die of a scrofulous affection of the abdominal glands before attaining the age of six years, respectively. He rejoined: "She has brought me six, but, sir, they are all under the sod, and all died as you have stated, and the cholera took her off, and I married again, and this miniature is a good likeness of my present wife, now sir, what do you think of my present prospects of viable children?" I responded, it is good, no man has better. He rejoined, by raising his feet, rubbing his hands, and exclaiming "I have three as fine boys as any body ever saw!"

This gentleman and his first wife were, respectively, *sanguine*, *bilious*, *lymphatic;* and his second wife was *bilious*.

In October, 1860, Mr. ——— and his wife called on me at my study. He said, "My wife and I have been informed that if you see a married couple you can tell what their luck will be as regards children, is it true, sir?" I responded: it is. He rejoined: "well sir, we have called to get you to tell us, if you please?" I answered: from your apparent ages, respectively, I think you have had your luck, and know more about it than I can tell you. He laughed and said: "you are right, sir, but we desire your opinion of what it has been?" I responded: you appear to be influenced by curiosity. He rejoined: "perhaps we are, sir, to some extent, but you will greatly oblige by giving us your opinion?" I responded: your luck has been of the worst possible character, for if your wife has had children, of which I am doubtful, three-fourths of them were dead-born and the others did not, respectively, live one year. I now addressed the wife: have you had children, madame? She answered: "yes, sir, eleven, and nine of them were dead-born, and the others lived only a few months.

I now addressed the husband: were not you and your wife sent here to try me? After a pause of hesitation, he answered: "yes, sir; two doctors, who have known us for many years to be sound and healthy, thought you could not tell what our luck had been, persuaded us to come and try you; I hope you will not blame us, for we meant no harm." I rejoined: as I have granted the favor you asked for, I desire you to do me a favor. He rejoined: "well, sir, what is it?" I answered: I wish you to say to those doctors, that I say they have not minds enough for doctors, but enough for chicken-thieves, and that for it they are mean enough. He answered: "I will do it, sir; for they ought not to have sent us here."

These parties were, respectively, *bilious encephalo-lymphatic*. He was more encephalic than she, and she more lymphatic than he; and, but for this difference, sterility would, probably, have been the result of their marriage.

In September, 1861, Mrs. ———, of Indiana, called on me said: "Prof. Powell, I have been, as I suppose, reliably informed that if you see a married couple, or their portraits, you can tell whether they are compatible or not; have I been correctly informed, sir?" I responded: you have madame. She rejoined: "when I obtained this information it interested me so much that I consulted several of my city physicians about it. They said, that they had heard of your pretensions and thought it to be an impossibility, and that you must be an extravagant humbug; but such was the source of my information that I did not yield to their opinions; and, as I had business up here, I resolved to bring my husband's daguerreotype and ascertain how the fact might be for myself. Here it is, sir. Now, if you please, your opinion of our marriage?" I responded: your marriage, I think, has been unfortunate, for if you have had children,—which is possible but scarcely probable,—they were either imbecile, died in early infancy of dropsy of the brain, or of a scrofulous variety of brain fever. She rejoined: "now, sir, I have the satisfaction of knowing that your pretension is not only possible but demonstrable. I am not, however, surprised that my physicians should have thought your pretensions impossible, but I am surprised that they should have so confidently expressed an opinion about a subject of which they certainly knew nothing." She resumed: "I have three children, sir, my first is still living, but my physician and neighbors say, that he is an idiot, but you say imbecile, pray, sir, what is the difference?" I explained. She rejoined: "then, sir, he is not an idiot, but an imbecile, my second died in the cradle of water on the brain, and my third, at about the same age, died of brain fever, but of what variety I never learned." She continued: "have I nothing to hope from my marriage better than I have had?" I answered: nothing except, probably, sterility. She added: "you have, beyond any doubt, made a discovery that will bless the world, and I rejoice to find you are more than equal to my most sanguine expectations."

These parties were respectively *bilious encephalic.* He was more encephalic than she, and she was more bilious than he; because of this difference children resulted. But believing that her maternally blighted hopes had increased, and was increasing, her encephalic condition, I thought it probable that sterility would result for her future.

In Oct., 1861, H——, Esq., called on me, from one of the lower counties of Kentucky, and said: "Professor POWELL, I have learned that if you see a married couple, or their portraits, you can tell whether they are fit for progenitors or not; is it true, sir?" I responded, it is. He rejoined: "It has always appeared to me to be reasonable, to suppose that human nature possessed the elements of the science of its most important function; the reproductive. Yet, as the literature of the day has not even furnished an intimation that such had been imagined as possible, and much less as having been discovered and developed into full blown reality, and hence I was sceptical about it, when informed of your pretension, and therefore I consulted several physicians about it, and with one exception your pretension was pronounced an impossibility, and you a humbug. But one of them said he 'had not heard of your pretension, but if you made such a pretension, he was inclined to believe you capable of the reality; that about twenty years ago, when you were a professor in the Medical College in New Orleans, he heard you lecture, and that you were then regarded as one of the most bold and original thinkers of the age. Sure I am, there is no humbug about him.' This, sir, encouraged me, and as I had business up here, I resolved to bring my wife's daguerreotype with me, and put you to the test of your pretension, provided you subject yourself to such tests." I rejoined: I do, sir, and am pleased to have them, because they furnish me new facts. He handed to me the daguerreotype with the interrogatory: "Are we fit for progenitors?" I responded: as you and she have a physiologically sound and healthy appearance, it is my opinion that in the abstract you are favorably constituted for progenitors, but in relation to each other, you are not. He rejoined, "what is the difficulty?" I answered, it is that your children will die of tabes mesenterica before attaining, respectively, the age of six years. He resumed: "You have got the prince of all the sciences; you have demonstrated the truth of all I have heard of you. A more sound and healthy couple than my wife and myself now are, and always have been, do not probably live, and yet, of the six children my wife has brought me, four have died as you have said they would, the fifth is going the same way, and the sixth is an infant and now appears promising. Must we lose it, too?" I answered, I think you will.

He continued: "My physicians insist that scrofula must have obtained in the ancestry of myself or my wife, but I am very confident that such was not the fact. What do you think of it, sir?" I responded: I know nothing of the ancestors of either you or your wife, and therefore have no opinion in relation to them. My conclusions in relation to your children were founded exclusively on my conception of the constitutions of you and your wife, respectively, and it was to the incompatibility between them that I attributed the scrofulous loss of your children, consequently I can conceive of no necessity for supposing scrofula to have obtained in the ancestry of either of you, but it may have, and therefore your physicians may be correct, for I know nothing about it, nor can I conceive of any necessity for the supposition. He resumed: "Would not this marriage incompatibility be a sufficient plea to either party for a divorce?" I answered: As it defeats the object of the marriage institution, it ought to be, and ultimately it will, I doubt not, but not now, I think. He rejoined, "but why not now?" I answered, because it is not known to our courts, physicians, or people. He continued: "Suppose I had brought you my wife's daguerreotype before we married; could you have informd me what would happen as you have what has happened?" I answered, the same, sir. He rejoined: "This subject is so important to the people that they will not long tolerate their physicians in an ignorance of it, consequently they will have to bestir themselves."

By the aid of this contribution, any physician who has but a very imperfect acquaintance with the temperaments can, in the circle of his own practice, in less than a day, satisfy himself of the truth of this discovery; because incompatible marriages and their consequences abundantly obtain in every town and city in our country. Physicians are now everywhere heard to say, that the children of some people cannot be cured when they become sick. Whose children are these? I answer: they are those of incompatible parents, for, invariably, if they escape imbecility, deafness, or blindness they have depraved constitutions.

The preceding parties were, respectively, *sanguine*, *bilious*, *lymphatic*, and very similar to the first parties in the second case,—and so were the results,—thus showing that the physiological, like the other natural laws, are uniform in their results.

I promptly respond to all questions, honestly propounded, in this relation. My observations in this relation for more than eighteen years have forced upon me the conviction that, physiologically, incompatible marriage is the remote cause of the scrofulous diathesis.

